# Deep dermatophytosis caused by *Trichophyton rubrum* in immunocompromised patients^⋆^

**DOI:** 10.1016/j.abd.2021.05.014

**Published:** 2022-01-20

**Authors:** Leandro Silva, João Sousa, Cristina Toscano, Isabel Viana

**Affiliations:** a Department of Dermatology, Hospital Egas Moniz, Centro Hospitalar Lisboa Ocidental, Lisboa, Portugal; b Microbiology Laboratory, Hospital Egas Moniz, Centro Hospitalar Lisboa Ocidental, Lisboa, Portugal

**Keywords:** Dermatofitose, Immunosuppression, Transplant, Trichophyton

## Abstract

In immunosuppressed patients, dermatophytosis can be more invasive, affecting the dermis and subcutaneous tissues. The authors describe the cases of two patients with kidney and heart transplanted, respectively, that developed a deep dermatophytosis caused by *Trichophyton rubrum*, confirmed by culture and DNA sequencing. Both patients had concomitant onychomycosis, and both were treated with itraconazole for about two months, which was interrupted due to pharmacological interactions with the immunosuppressive drugs and switched to terbinafine, leading to clinical resolution within four months. Deep dermatophytosis should be considered when dealing with immunocompromised patients, especially when a superficial dermatophytosis is present. Oral treatment is necessary and terbinafine is a preferable option in solid organ transplant recipients because it has less pharmacological interactions.

We described two cases: the first patient is a 55-year-old woman with a kidney transplanted seven months before that presented with erythematous plaques distributed on both legs (Fig. 1) and several violaceous papular lesions on the dorsum of the left foot and toes (Fig. 2). All cutaneous lesions were present for six months.

**Figure 1:**
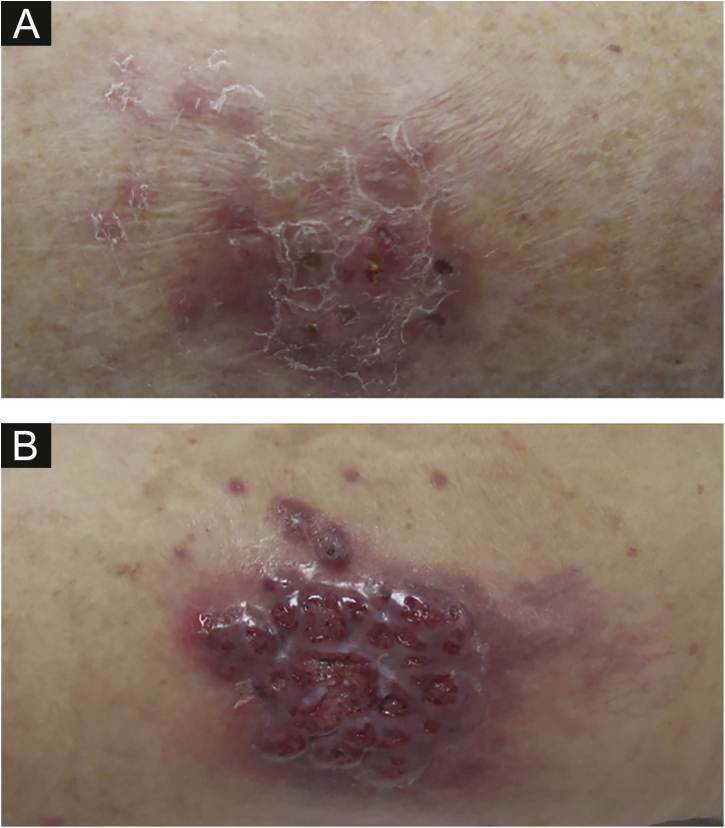
(A), First patient – Erythematous scaly plaque on the right leg. (B), Second patient – Violaceous granulomatous nodule surrounded by small erythematous papules on the left thigh.

**Figure 2:**
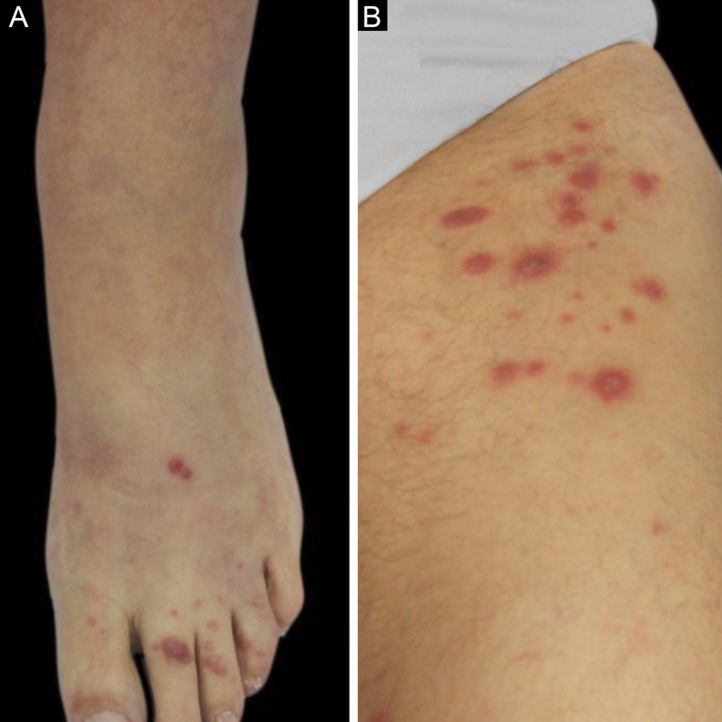
(A), First patient – Violaceous papular lesions on the dorsum of the left foot and left toes. (B), Second patient – Erythematous grouped papules on the superior portion of the left thigh.

The second patient is a 59-year-old woman who was submitted to a heart transplant four months prior to the onset of a painful violaceous granulomatous lesion on the left thigh (Fig. 1). At the time of consultation, this lesion had two months of evolution and since then, more nodular lesions have appeared on both limbs. She also had papular lesions on the thighs and pubic area (Fig. 2).

Both patients were under immunosuppressive therapy with tacrolimus and mycophenolate mofetil, and trimethoprim/sulfamethoxazole and prednisolone.

In both cases, onychomycosis of the toenails was present. In each case, skin biopsies were performed for routine histology and mycological examination.

Both biopsies showed multiple suppurative granulomas, consisting of numerous neutrophils surrounded by histiocytes and giant cells. Fungal septate hyphae were positive with Periodic AcidSchiff (PAS) and Grocott’s methenamine silver staining (GMS) (Figure 3, Figure 4).

**Figure 3:**
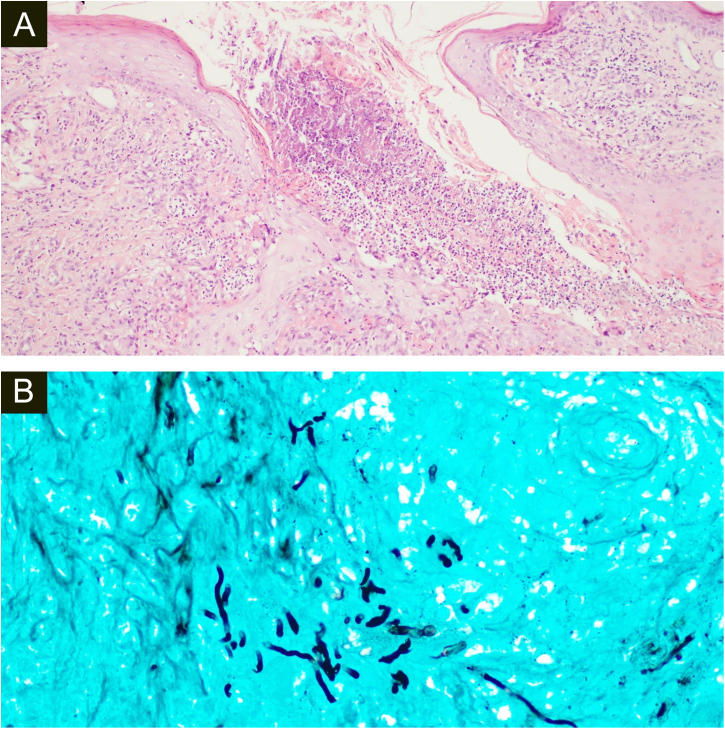
(A), Histopathology of the surgical specimen of the first patient shows diffuse oedema and mixed inflammatory cells with formation of micro abscesses the hyaline, hyphae in multinucleated giant cells with surrounding neutrophils (Hematoxylin & eosin, ×100). (B), Positive Grocott’s methenamine silver stain with multiple branching septate hyphae (×400).

**Figure 4:**
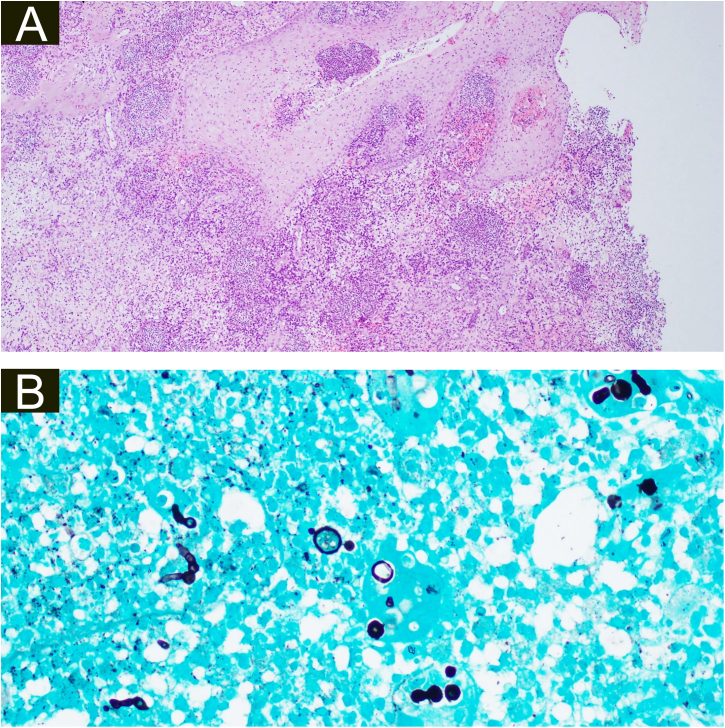
(A), Histopathology of the surgical specimen of the second patient demonstrating pseudoepitheliomatous hyperplasia, exocytosis of neutrophils and lymphocytes, diffuse oedema mixed inflammatory cells with formation of micro abscesses in the dermis and dermal capillary proliferations, with fungal hyphae (basophilic globular structures) in the multinucleated giant cells (Hematoxylin & eosin, ×100). (B), Grocott’s methenamine silver stain branching septate hyphae and multiple round yeast-like structures with pseudobudding (×400).

In both patients, culture for mycology examination was performed on Sabouraud’s Dextrose Agar (SDA) and *Trichophyton rubrum (T. rubrum)* was isolated (Figure 5, Figure 6). Identification was confirmed by sequencing of ribosomal DNA (GenBank accession number MK967277) and scraping from toenails also isolated *T. rubrum*.

**Figure 5:**
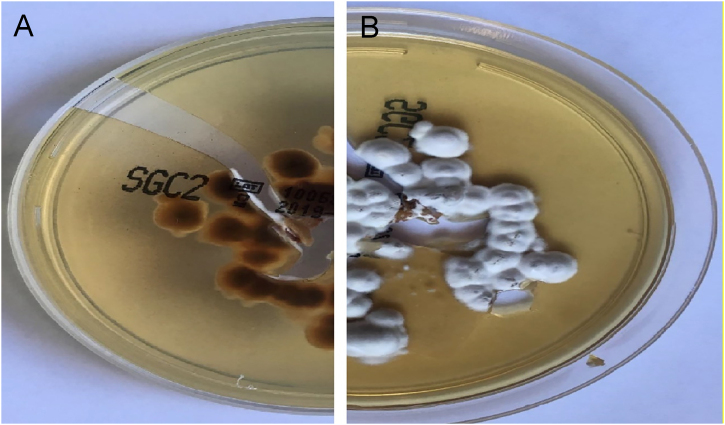
Sabouraud’s Dextrose Agar culture of the first patient. (A), Reverse of colony with a yellow-red color. (B), Front view with white powdery colonies. These aspects are suggestive of *T. rubrum.*

**Figure 6:**
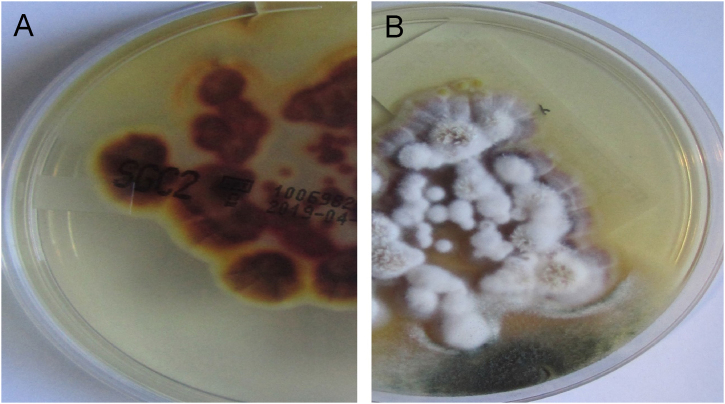
Second patient Sabouraud’s Dextrose Agar culture. (A), Reverse of colony with a yellow-brown color. (B), Front view with white powdery colonies. These findings are compatible with *T. rubrum.*

Assuming a diagnosis of deep dermatophytosis to *T. rubrum*, itraconazole 200 mg daily was initiated. For the next two months, there was difficulty in maintaining tacrolimus levels because of pharmacological interaction with itraconazole. Hence, itraconazole was stopped and terbinafine 250 mg/day initiated, resulting in clinical improvement, four months after therapy switch, with consequent healing of all lesions and no recurrence after treatment interruption.

Dermatophytosis are fungal infections typically confined to keratinized tissues.^1^ The most frequent causal dermatophyte worldwide is *T. rubrum.*^1^

In immunocompromised patients, dermatophytosis can be more invasive, affecting the dermis and subcutaneous tissues.^1^, ^2^

Invasive dermatophytosis can be classified as Majocchi’s granuloma, deep dermatophytosis and disseminated dermatophytosis.^1^, ^2^, ^3^, ^4^ The distinction between Majocchi’s granuloma and deep dermatophytosis is that the latter is not restricted to perifollicular areas.^2^, ^3^, ^4^

There are less than 100 cases of deep dermatophytosis reported in the literature, mainly occurring in solid organ transplant recipients.^2^, ^4^, ^5^

Clinically, deep dermatophytosis manifests usually as multiple papules, plaques, and nodules, most frequently located on the lower extremities.^4^, ^5^, ^6^, ^7^ Diagnosis should be considered in immunosuppressed patients, particularly when there are associated superficial dermatophytosis, such as onychomycosis.^7^, ^8^ In the present cases, both patients had concomitant onychomycosis of the toenails caused by *T. rubrum*.

Confirmation of the infection requires visualization of hyphae in the dermis and identification of the microorganisms in culture or by DNA sequencing.^7^, ^8^

There is no consensus on the best treatment, but systemic antifungal therapy with terbinafine or itraconazole is usually effective.^5^, ^9^ Rouzaud et al. proposed terbinafine as first-line therapy.^5^

Given the pharmacological interactions between itraconazole and other immunosuppressive medication, we would also advocate for terbinafine as first-line therapy for deep dermatophytosis.

## Financial support

None declared.

## Authors’ contributions

Leandro Silva: Study concept and design; data collection, or analysis and interpretation of data; writing of the manuscript or critical review of important intellectual content; Data collection, analysis and interpretation; Intellectual participation in the propaedeutic and/or therapeutic conduct of the studied cases; Critical review of the literature; Final approval of the final version of the manuscript.

João Sousa: Data collection, analysis and interpretation; Intellectual participation in the propaedeutic and/or therapeutic conduct of the studied cases.

Cristina Toscano: Data collection, analysis and interpretation; Intellectual participation in the propaedeutic and/or therapeutic conduct of the studied cases.

Isabel Viana: Writing of the manuscript or critical review of important intellectual content; Final approval of the final version of the manuscript.

## Conflicts of interest

None declared.
